# Retraction: Sports resources utilization efficiency, productivity change, and regional production technology heterogeneity in Chinese Provinces: DEA-SBM and Malmquist Index Approaches

**DOI:** 10.1371/journal.pone.0352816

**Published:** 2026-07-08

**Authors:** The PLOS One Editors

The *PLOS One* Editors retract this article [1] because it was identified as one of a series of submissions for which we have concerns about compromised peer review and compliance with PLOS policy on Manipulation of the Publication Process.

In addition, the articles cited as References 6 and 7 were retracted during the peer review of [1]; the article cited as Reference 78 was retracted after acceptance but prior to publication of [1]; and the articles cited as References 41 and 81 were retracted after publication of [1].

HZ, XJ, XX, and WUHS did not agree with the retraction. YS either did not respond directly or could not be reached.
